# Association between *Cyclin D1* G870A (rs9344) polymorphism and cancer risk in Indian population: meta-analysis and trial sequential analysis

**DOI:** 10.1042/BSR20180694

**Published:** 2018-11-30

**Authors:** Nisha Thakur, Suchitra Kumari, Ravi Mehrotra

**Affiliations:** 1 Division of Molecular Diagnostics, National Institute of Cancer Prevention and Research (NICPR)ICMR, I-7, Sector-39, Noida, Gautam Buddha Nagar, Uttar Pradesh 201301, India; 2 Data Management Laboratory, National Institute of Cancer Prevention and Research (NICPR)ICMR, I-7, Sector-39, Noida, Gautam Buddha Nagar, Uttar Pradesh 201301, India; 3 Division of Preventive Oncology, National Institute of Cancer Prevention and Research (NICPR)ICMR, I-7, Sector-39, Noida, Gautam Buddha Nagar, Uttar Pradesh 201301, India

**Keywords:** CCND1, G870A, India, Meta-analysis, Polymorphism, rs9344

## Abstract

**Introduction:** Association between Cyclin D1 (CCND1) single nucleotide polymorphism (SNP) rs9344 and cancer risk is paradoxical. Thus, we performed a meta-analysis to explore the association between *CCND1* variant and overall cancer risk in Indian population. **Methods:** Data from 12 published studies including 3739 subjects were collected using *Pubmed* and *Embase. RevMan (Review Manager) 5.3* was used to perform the meta-analysis. OR with 95%CI were calculated to establish the association. **Results:** Overall, the cumulative findings demonstrated that *CCND1* polymorphism (rs9344) was not significantly associated with cancer risk in all the genetic models studied (dominant model: GG vs GA+AA: OR (95%CI) = 0.81 (0.60–1.09), *P*=0.17; recessive model: GG+GA vs AA: OR (95%CI) = 1.23 (0.96–1.59), *P*=0.11; co-dominant model: **GG vs AA: OR (95%CI) = 1.35 (0.93–1.97), *P*=0.12;** co-dominant model: **(GG vs GA: OR (95%CI) = 1.16 (0.85–1.59), *P*=0.34**; allelic model: A vs G: OR (95%CI) = 1.20 (1.14–2.85), *P*=0.23; allelic model: G vs A: OR (95%CI) = 0.83 (0.62–1.12), *P*=0.23). Subgroup analysis according to cancer types presented significant association of *CCND1* polymorphism and increased breast cancer risk in dominant model (GG vs GA+AA: OR = 2.75, 95%CI = 1.54–4.90, *P*=0.0006) and allelic model (G vs A: OR = 1.63, 95%CI = 1.22–2.19, *P*=0.001). An increased esophageal cancer risk in recessive model (GG+GA vs AA: OR = 1.51, 95%CI = 1.05–2.16, *P*=0.03) and co-dominant model (GG vs AA: OR = 2.51, 95%CI = 1.10–5.71, *P*=0.03) was detected. A higher risk for colorectal cancer was detected under both the co-dominant models (GG vs AA: OR = 2.46, 95%CI = 1.34–4.51, *P*=0.004 and GG vs GA: OR = 1.74, 95%CI = 1.14–2.67, *P*=0.01). However, in case of cervical cancer risk a non-significant association was reported under the recessive model (GG+GA vs AA: OR = 1.52, 95%CI = 0.60–3.90, *P*=0.38) with reference to *CCND1* polymorphism (rs9344). The trial sequential analysis (TSA) showed that the cumulative Z-curve neither crossed the trial sequential monitoring boundary nor reached the required information size (RIS). Thus, present meta-analysis remained inconclusive due to insufficient evidence. **Conclusion:** *CCND1* polymorphism rs9344 may not have a role in overall cancer susceptibility in Indian population. However, this polymorphism acts as a crucial risk factor for breast, esophageal, and colorectal cancer but not for cervical cancer. Future studies with larger sample size are required to draw a reliable conclusion.

## Introduction

Cancer is a major global health problem and it is worse in case of low- and middle-income developing countries. According to India’s National Cancer Registry Program (NCRP), 1.45 million cases would occur in 2016 with 0.74 million deaths in India. This is expected to rise to 1.73 million cases and 0.88 million deaths in 2020 [1,2]. Cancer is considered the disease of abnormal cell division. Besides, many environmental cofactors (smoking, use of alcohol, exposure to UV radiations, infections with certain viruses) and host genetic makeup has been recognized as a pivotal risk factor for human cancers.

India ranks third in the world in terms of incidence rate of cancer cases amongst women after China and the U.S.A. According to the Globocan report 2012, there were ∼232000 breast cancer cases registered in the U.S.A., however in India, 145000 new cases were reported. The burden of breast cancer in India is approximately two-thirds of that of the U.S.A. and is growing progressively [3]. Breast cancer is one of the most common malignancies in women worldwide, and each year more than 1 million new cases are diagnosed [4]. The main risk factors for breast cancer are genetic predisposition, lifestyle, and environment [5–7]. Genetic polymorphisms have been identified as one of the crucial factor for determining inter-individual susceptibility to cancer [8]. The clinical importance of *CCND1* gene lies in the fact that 5–20% of breast cancer cases present with either amplified or deleted version of the gene [9,10]. *CCND1* also has documented oncogenic characteristics by manipulating the regulation of cell cycle machinery particularly at the transition phase of G_1_/S [11,12]. Cyclin D1 (CCND1) protein is found to be overexpressed in more than 50% of breast cancer cases [13]. An important functional single nucleotide polymorphism (SNP) in *CCND1* gene (rs9344) G870A, may influence the breast cancer development [14]. Esophageal cancer is the eighth most common cancer overall. In 2012, worldwide, 456000 new cases have been estimated (3.2% of all incidence cancer cases). It is the sixth most common cause of death from cancer, with an estimated 400000 deaths in 2012 (4.9% of all cancer deaths) [3]. It is one of the most common and lethal type of cancer worldwide, with <20% of 5-year survival rate [15]. Colorectal cancer is the third most commonly diagnosed cancer in men and second in women, with >1.4 million new cases annually [16]. Geographical deviation in the incidence rates has been observed as developed world contributes to >50% of the cases. Though, mortality is more in the developing countries due to insufficient resources and health infrastructure [17]. In India, the age standardized rate (ASR) for colorectal cancer is 7.2 per 100000 men and 5.1 per 100000 women [3].

*CCND1* is a key cell cycle regulatory gene which governs the G_1_/S checkpoint in cell cycle. It is one of the most frequently altered molecules in human carcinogenesis. A common G/A SNP [dbSNP ID rs9344] was first described by Betticher et al. (1995) [18]. This SNP rs9344 is located at codon 242 in the exon-4/intron boundary of *CCND1* and responsible for alternate splicing of transcripts with different half-lives [18]. Since then many case–control studies have been conducted to explore the potential association between *CCND1* SNP (rs9344) and cancer susceptibility. Occurrence of this nucleotide variation has been found to be coupled with the risk of various cancers including cervical, breast, oral, esophageal, lung, urinary bladder, prostate, and colorectal [19–29]. The outcomes of these studies were inconsistent in different ethnic groups. To overcome this conflict, several meta-analyses have been performed worldwide to see the effect of *CCND1* polymorphism and risk for different types of cancer [30–35]. To the best of our knowledge, no report is available from India addressing the impact of *CCND1* SNP and overall cancer risk. Hence, we aimed to investigate the role of *CCND1* polymorphism G870A (rs9344) in overall cancer susceptibility amongst Indian population by conducting this meta-analysis. The present data could be helpful in enriching the existing knowledge with respect to involvement of *CCND1* polymorphism and cancer susceptibility in Indian population.

## Methods

### Literature search strategy

*Pubmed* and *Embase* databases were searched with the keywords ‘*CCND1*’, ‘Cyclin D1’, ‘SNP’, ‘cancer’, ‘India’, and ‘polymorphism’ for literature published till September 2016. All studies included in the present meta-analysis met the following inclusion and exclusion criteria.

### Inclusion criteria

(i) Prospective or case–control studies involving association analysis between *CCND1* SNP G870A (rs9344) and cancer susceptibility, (ii) studies included Indian population, (iii) genotypic and allelic details are provided for both the cases and control groups, (iv) full text available, and (v) articles published in English language.

### Exclusion criteria

(i) Studies published on populations other than Indian, (ii) articles published in languages except English, and (iii) articles not providing genotypic and allelic details.

### Data retrieval

Data from all eligible studies were retrieved independently by two investigators (N.T. and S.K.). The retrieved data incorporated the following details: (i) PubMed IDentifier (PMID), (ii) name of the first author, (iii) year of publication, (iv) country, (v) sources of controls, (vi) methods for genotyping, and (vii) frequency of genotypic and allelic data.

### Quality assessment

Quality of the included studies was assessed by assigning the quality scores as previously mentioned by He et al. (2014) [36]. The scores were assigned to each qualified studies between 0 and 10. Studies with >5 scores were included for the further analyses (Supplementary Table S1).

### Meta-analysis

RevMan (*Review Manager)* is an easy tool to perform the meta-analyses and generate the graphs (forest plot, funnel plot) in publication standard. Meta-analysis of *CCND1* gene G870A polymorphism (rs9344) was performed by *RevMan 5.3* [37]*.* For statistical models, both fixed model and random model were included in the RevMan. For random models, DerSimonian and Laird random-effects models were used. Odds ratios (ORs) with 95% confidence intervals (95%CIs) were used to assess the strength of association between the *CCND1*-G870A polymorphisms and cancer risk. The pooled OR was evaluated by the Z-test and a *P*-value <0.05 suggests a significant association.

*I^2^* was used to estimate total variation across studies due to heterogeneity in percentage. A percentage of <25% was considered as a low level of heterogeneity, 25–50% as a moderate level of heterogeneity, and >50% as a high level of heterogeneity. *I^2^*> 50% could suggest heterogeneity and suggest using a random-effect estimate [38]. Otherwise, the fixed-effect model was used to calculate pooled ORs [39].

Software RevMan 5.3 used in this meta-analysis is freely available at http://community.cochrane.org/tools/review-production-tools/revman-5/revman-5-download

### Statistical analysis

The association between *CCND1* polymorphism and cancer risk was analyzed by OR with 95%CI in different genetic models: dominant (GA+AA vs GG), recessive (AA vs GG+GA), co-dominant (GA vs GG and AA vs GG), and allelic (A vs G and G vs A). The *P*-value <0.05 was considered statistically significant. Subgroup analysis was done after stratification of data according to various cancer types.

Heterogeneity was calculated by chi-square test and the extent of heterogeneity was measured by the value of *I^2^* statistic. The OR of different types of genetic models was evaluated by employing the fixed-effect model (when *I^2^* < 50%) or random-effect model (when *I^2^* > 50%). Egger’s bias test and Begg’s funnel plot was used to assess the publication bias [40,41]. It is a well-acknowledged fact that meta-analyses are vulnerable to random errors due to sparse data and repetitive testing of accrued data [42]. Hence, trial sequential analysis (TSA) was performed to minimize the type I error and random error as the present study had smaller sample size. TSA was performed as described previously by Fu et al. (2017) [43]. It was done by using TSA software version 0.9.5.10. (http://www.ctu.dk/tsa/) [44] to calculate the required information size (RIS) (meta-analysis sample size) by taking the control event proportion to 25.77%, experimental event proportion 21.55%, a relative risk reduction (RRR) 10%, power 80%, and type I error (α) 5%. The monitoring boundaries were constructed to determine whether present meta-analysis is sufficiently powered and conclusive. Therefore, it is able to reject false-positive reports from meta-analysis [45]. If the Z-curve crosses the TSA boundaries or futility area, there is sufficient information to support the conclusions and further trials are unlikely to change the findings. If the Z-curve does not cross the any of the boundaries or reach the RIS, evidence is insufficient to reach a firm conclusion.

## Results

### Study characteristics

**Figure 1:**
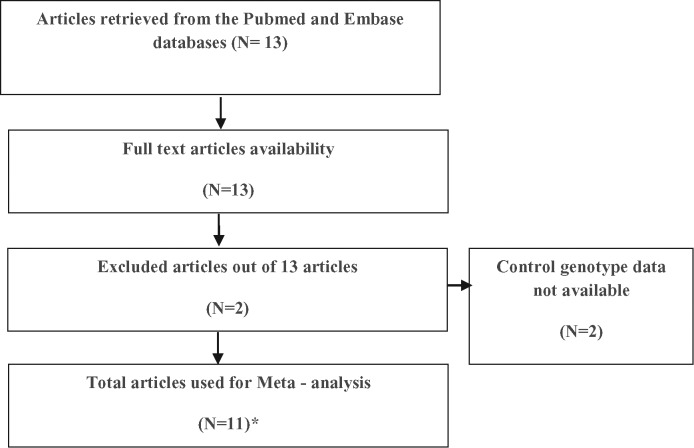
Methodology flowchart for the selection of studies in the present meta-analysis *Since, data from study PMID 24604328 were extracted twice, hence total articles mentioned are 12 in the text.

Using the *Pubmed* and *Embase* database, a total of 12 studies were searched independently by two investigators (S.K. and N.T.) according to the methodology depicted in flow diagram (Figure 1).

Data from one study with PMID 24604328 was extracted twice. All the 12 studies including 1791 cancer cases and 1948 controls met our inclusion criteria. The characteristics of included studies for the present meta-analysis from different cancers are presented in Table 1.

**Table 1:** Characteristics of the studies included in the meta-analysis

S.No.	PMID	Authors	Publication year	Country	Ethnicity	Source of control	Cancer type	Genotyping methods
1.	16488657	Sathyan et al. [22]	2006	India	Asian	Hospital based	Oral cancer	PCR-SSCP
2.	17011980	Sobti et al [25]	2006	India	Asian	Hospital based	Lung cancer	PCR
3.	17561354	Jain et al. [23]	2007	India	Asian	Hospital based	Esophageal cancer	PCR-RFLP
4.	18548202	Kaur et al. [20]	2008	India	Asian	Hospital based	Cervical cancer	PCR-RFLP
5.	19489683	Thakur et al. [19]	2009	India	Asian	Hospital based	Cervical cancer	PCR-RFLP
6.	20380574	Gangwar et al. [26]	2010	India	Asian	Hospital based	Urinary bladder cancer	PCR-RFLP
7.	21268129	Hussain et al. [24]	2011	India	Asian	Hospital based	Esophageal squamous cell carcinoma	PCR-RFLP
8.	20822933	Mandal et al. [27]	2012	India	Asian	Hospital based	Prostate cancer	PCR-RFLP
9.	23354584	Sameer et al. [29]	2013	India	Asian	Hospital based	Colorectal cancer	PCR-RFLP
10.	24604328	Wasson et al. [21]	2014	India	Asian	Hospital based	Breast cancer	PCR-RFLP
11.	24604328*	Wasson et al. [21]	2014	India	Asian	Hospital based	Breast cancer	PCR-RFLP
12.	25146682	Govatati et al. [28]	2014	India	Asian	Hospital based	Colorectal cancer	PCR

Abbreviation: RFLP, restriction fragment length polymorphism. *PMID24604328 taken twice.

Details of genotypic and allelic frequencies of *CCND1* polymorphism is shown in Table 2.

**Table 2:** Distribution of CCND1-G870A genotypes and allelic frequency in cancer cases and controls

S.No.	PMID	Cancer type	Case	Control	Case			Control			Case		Control	
			*n*	*n*	GG	GA	AA	GG	GA	AA	A	G	A	G
1.	16488657	Oral cancer	146	137	36	71	39	40	61	36	0.51	0.49	0.49	0.51
2.	17011980	Lung cancer	151	151	29	87	35	39	69	43	NA	NA	NA	NA
3.	17561354	Esophageal cancer	151	201	22	76	53	37	111	53	NA	NA	NA	NA
4.	18548202	Cervical cancer	150	150	33	64	53	30	65	55	NA	NA	NA	NA
5.	1948683	Cervical cancer	200	200	39	94	67	47	119	34	228	172	187	213
6.	20380574	Urinary bladder cancer	212	250	48	85	79	58	119	73	243	181	265	235
7.	20822933	Prostate cancer	192	224	38	65	89	58	93	73	243	141	239	209
8.	21268129	Esophageal cancer	151	151	20	99	32	56	72	23	163	139	118	184
9.	23354584	Colorectal cancer	130	160	19	70	41	41	76	43	NA	NA	NA	NA
10.	24604328	Breast cancer	151	83	33	77	41	07	47	29	159	143	105	61
11.	24604328*	Breast cancer	54	134	15	31	08	18	78	38	47	61	154	114
12.	25146682	Colorectal cancer	103	107	54	39	10	71	33	03	59	147	39	175

* PMID: 24604328 repeated twice in our study. NA, not available.

### Meta-analysis of CCND1 G/A polymorphism (rs9344)

**Figure 2:**
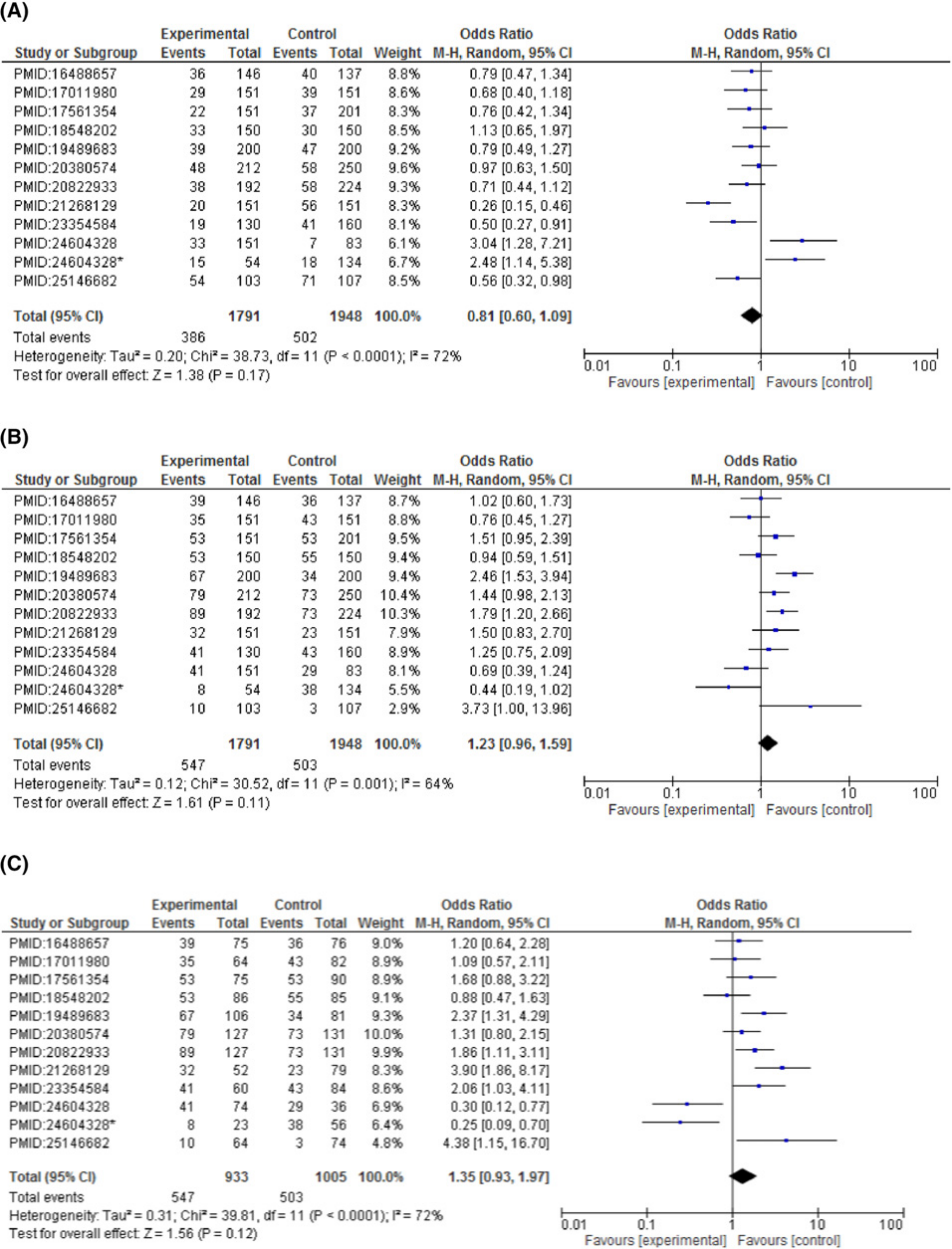

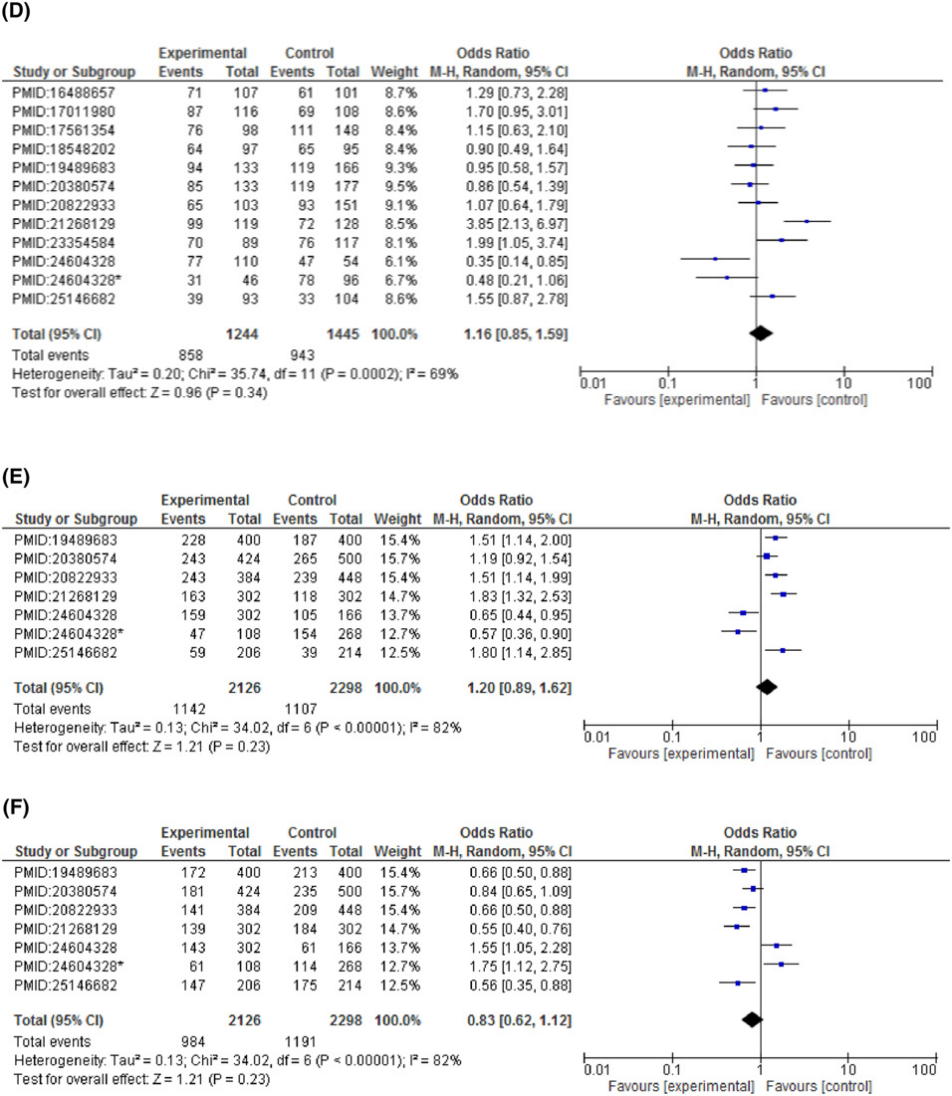
Forest plots describing the association of *CCND1*-G870A polymorphism with overall cancer risk (**A**) dominant model (GG vs GA+AA); (**B**) recessive model (GG+GA vs AA); (**C**) co-dominant model (GG vs GA); (**D**) co-dominant model (GG vs AA); (**E**) allele model (A vs G); (**F**) allele model (G vs A).

A total of 12 studies were included in the analysis to evaluate the association between *CCND1* polymorphism and cancer risk in Indian population. The results from meta-analysis of the association between *CCND1* polymorphism (rs9344) and cancer risk in 12 case–control studies are shown in Figure 2 and Table 3. Values of ORs with 95%CI were as follows: dominant model (GG vs GA+AA: OR = 0.81, 95%CI = 0.60–1.09, *P*=0.17, *I^2^*= 72%); recessive model (GG+GA vs AA: OR = 1.23, 95%CI = 0.96–1.59, *P*=0.11, *I^2^* = 64%); co-dominant model (GG vs AA: OR = 1.35, 95%CI = 0.93-1.97, P = 0.12, I^2^ = 72%); co-dominant model (GG vs GA: OR = 1.16, 95%CI = 0.85–1.59, *P*=0.34, *I^2^* = 69%); allele model (A vs G: OR = 1.20, 95%CI = 1.14–2.85, *P*=0.23, *I^2^* = 82%), and allele model (G vs A: OR = 0.83, 95%CI = 0.62–1.12, *P*=0.23, *I^2^* = 82%) (Table 3). If the values of *I^2^* were >50% then the random-effect model was applied, otherwise fixed-effect model was used to calculate the pooled ORs and 95%CI. In meta-analysis, *P^Z^*<0.05 was considered statistically significant. Here, we demonstrate that *CCND1* polymorphism G870A (rs9344) is not associated with the risk for overall cancers in Indian population.

**Table 3:** Meta-analysis results based on different genetic models

S.No.	Category	OR	[95%CI]	*P*^*Z*^	*P^H^*	*I^2^* (%)	Statistical method
1.	Dominant model (GG vs GA+AA)	0.81	[0.60–1.09]	0.17	<0.0001	72%	Random
2.	Recessive model (GG+GA vs AA)	1.23	[0.96–1.59]	0.11	0.001	64%	Random
3.	Co-dominant model (AA vs GG)	1.35	[0.93, 1.97]	0.12	<0.0001	72%	Random
4.	Co-dominant model (GA vs GG)	1.16	[0.85, 1.59]	0.34	0.0002	69%	Random
5.	Allele model (A vs G)	1.20	[1.14–2.85]	0.23	<0.00001	82%	Random
6.	Allele model (G vs A)	0.83	[0.62–1.12]	0.23	<0.00001	82%	Random

Abbreviations: P^H^, P value for heterogeneity; P^Z^, P value for Z-test.

**Figure 3:**
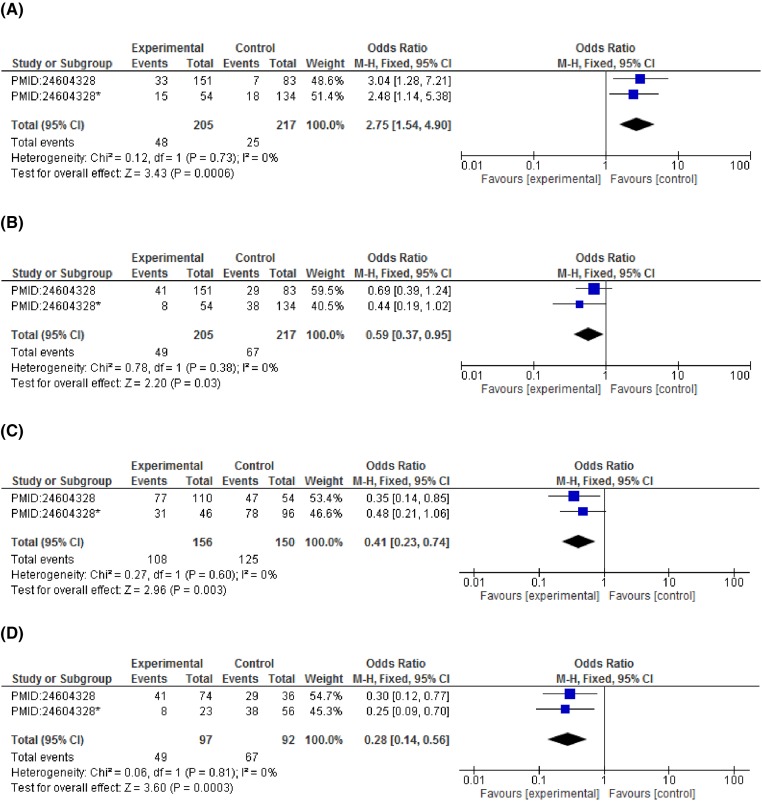

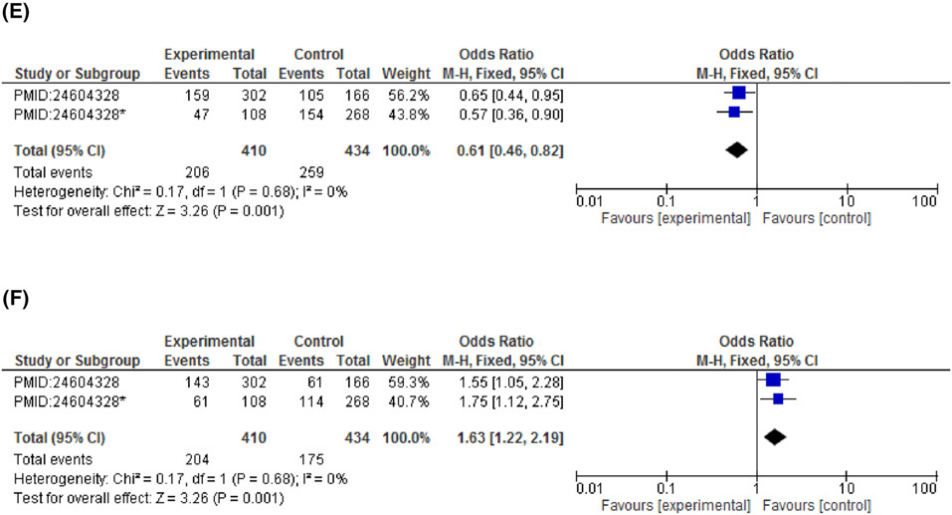
Forest plots describing the association of *CCND1*-G870A polymorphism with breast cancer risk (**A**) dominant model (GG vs GA+AA); (**B**) recessive model (GG+GA vs AA); (**C**) co-dominant model (GG vs GA); (**D**) co-dominant model (GG vs AA); (**E**) allele model (A vs G); (**F**) allele model (G vs A).

**Figure 4:**
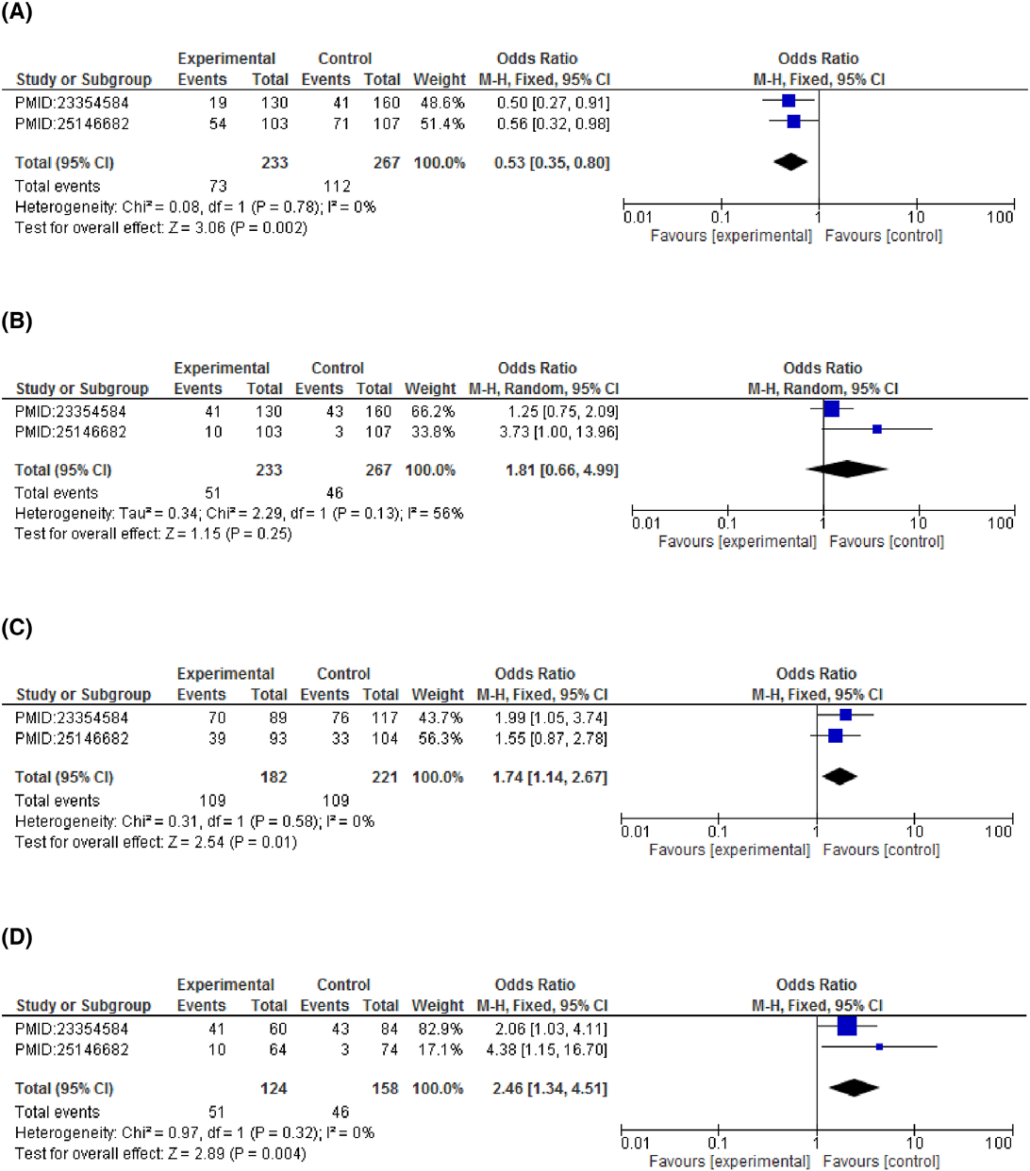
Forest plots describing the association of *CCND1-G870A* polymorphism with colorectal cancer risk (**A**) dominant model (GG vs GA+AA); (**B**) recessive model (GG+GA vs AA); (**C**) co-dominant model (GG vs GA); (**D**) co-dominant model (GG vs AA).

**Figure 5:**
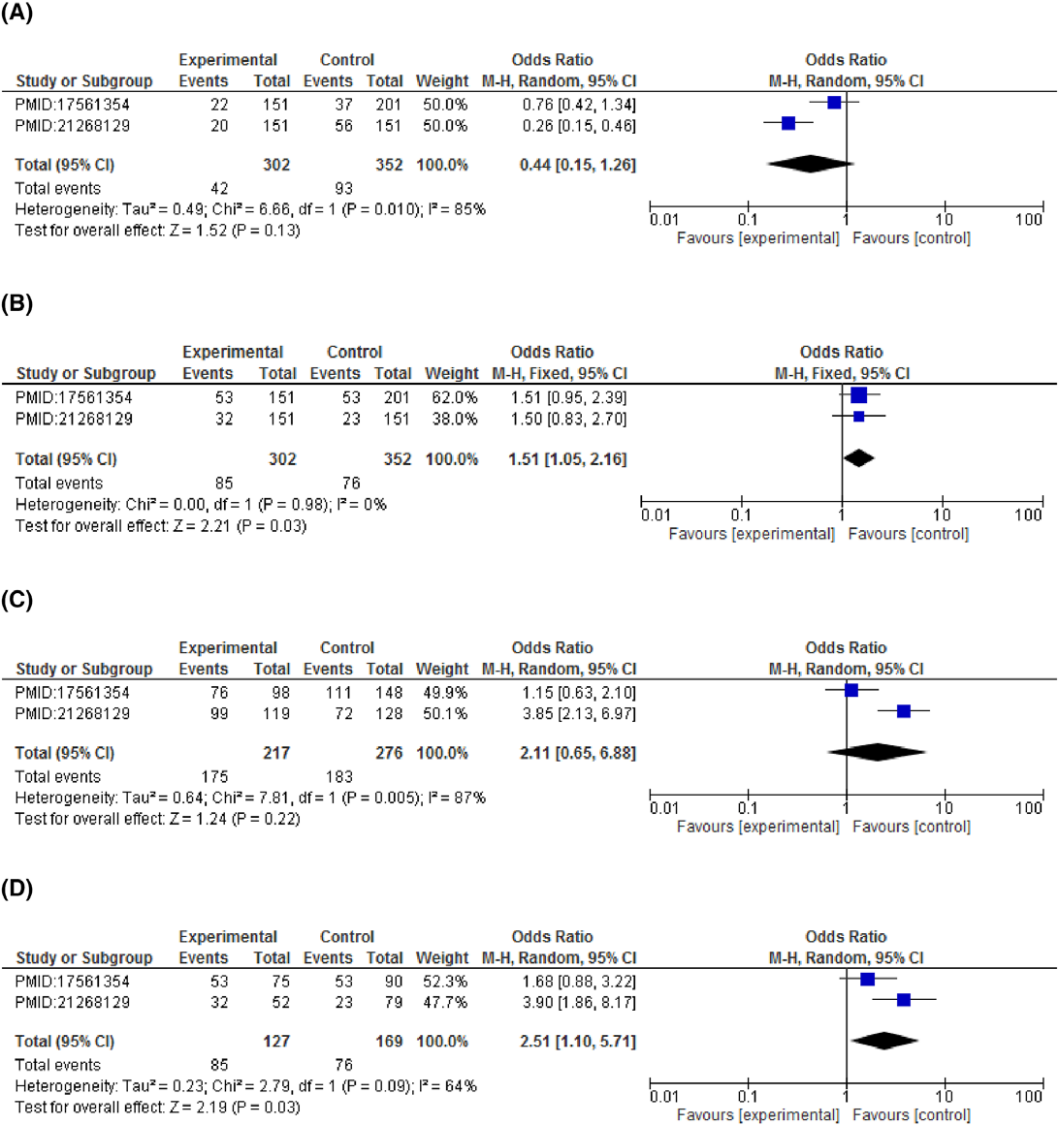
Forest plots describing the association of *CCND1*-G870A polymorphism with esophageal cancer risk (**A**) dominant model (GG vs GA+AA); (**B**) recessive model (GG+GA vs AA); (**C**) co-dominant model (GG vs GA); (**D**) co-dominant model (GG vs AA).

**Figure 6:**
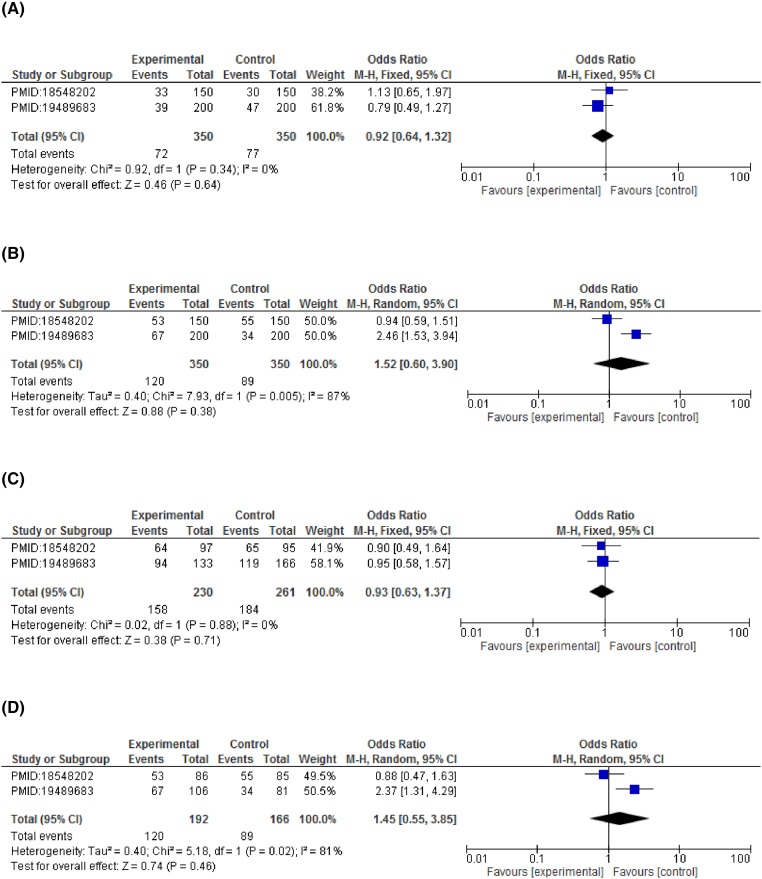
Forest plots describing the association of *CCND1*-G870A polymorphism with cervical cancer risk (**A**) dominant model (GG vs GA+AA); (**B**) recessive model (GG+GA vs AA) (**C**) co-dominant model (GG vs GA); (**D**) co-dominant model (GG vs AA).

On subgroup analysis stratified according to cancer types showed significant association of *CCND1* polymorphism and increased breast cancer risk in dominant model (GG vs GA+AA: OR = 2.75, 95%CI = 1.54–4.90, *P*=0.0006), allelic model (G vs A: OR = 1.63, 95%CI = 1.22–2.19, *P*=0.001). A statistically significant association with esophageal cancer risk was observed in recessive (GG+GA vs AA: OR = 1.51, 95%CI = 1.05–2.16, *P*=0.03) and co-dominant model (GG vs AA: OR = 2.51, 95%CI = 1.10–5.71, *P*=0.03). An increased risk for colorectal cancer was detected under both the co-dominant models (GG vs AA: OR = 2.46, 95%CI = 1.34–4.51, *P*=0.004 and GG vs GA: OR = 1.74, 95%CI = 1.14–2.67, *P*=0.01). Contrary to this, none of the genetic model reported a statistically significant association with cervical cancer risk. Although a non-significant association was observed in recessive model (GG+GA vs AA: OR = 1.52, 95%CI = 0.60–3.90, *P*=0.38) and co-dominant model (GG vs AA: OR = 1.45, 95%CI = 0.55–3.85, *P*=0.46) with reference to *CCND1* polymorphism (rs9344) (Figures 3–6 and Table 4).

**Table 4:** Subgroup analysis: meta-analysis results according to the type of cancer

Subgroup	OR	(95%CI)	*P^Z^*	*P*^*H*^	I^*2*^ (%)	Effects model
**Breast cancer**						
Dominant model (GG vs GA+AA)	2.75	(1.54–4.90)	**0.0006**	0.73	0%	Fixed
Recessive model (GG+GA vs AA)	0.59	(0.37–0.95)	**0.03**	0.38	0%	Fixed
Co-dominant model (GG vs GA)	0.41	(0.23–0.74)	**0.003**	0.60	0%	Fixed
Co-dominant model (GG vs AA)	0.28	(0.14–0.56)	**0.0003**	0.81	0%	Fixed
Allele model (A vs G)	0.61	(0.46–0.82)	**0.001**	0.68	0%	Fixed
Allele model (G vs A)	1.63	(1.22–2.19)	**0.001**	0.68	0%	Fixed
**Colorectal cancer**						
Dominant model (GG vs GA+AA)	0.53	(0.35–0.80)	**0.002**	0.78	0%	Fixed
Recessive model (GG+GA vs AA)	1.81	(0.66–4.99)	0.25	0.13	56%	Random
Co-dominant model (GG vs GA)	1.74	(1.14–2.67)	**0.01**	0.58	0%	Fixed
Co-dominant model (GG vs AA)	2.46	(1.34–4.51)	**0.004**	0.32	0%	Fixed
**Esophageal cancer**						
Dominant model (GG vs GA+AA)	0.44	(0.15–1.26)	0.13	0.010	85%	Random
Recessive model (GG+GA vs AA)	1.51	(1.05–2.16)	**0.03**	0.98	0%	Fixed
Co-dominant model (GG vs GA)	2.11	(0.65–6.88)	0.22	0.005	87%	Random
Co-dominant model (GG vs AA)	2.51	(1.10–5.71)	**0.03**	0.09	64%	Random
**Cervical cancer**						
Dominant model (GG vs GA+AA)	0.92	(0.64–1.32)	0.64	0.34	0%	Fixed
Recessive model (GG+GA vs AA)	1.52	(0.60–3.90)	0.38	0.005	87%	Random
Co-dominant model (GG vs GA)	0.93	(0.63–1.37)	0.71	0.88	0%	Fixed
Co-dominant model (GG vs AA)	1.45	(0.55–3.85)	0.46	0.02	81%	Random

Abbreviations: *P^Z^, P*-value for Z-test; *P^H^, P*-value for heterogeneity. Statistically significant values shown in bold.

### Heterogeneity measurement

Heterogeneity value depicted as *I^2^* was calculated for different genetic models and presented in Table 3. Heterogeneity was observed in all the genotypic and allelic models. For dominant model: GG vs GA+AA: *I^2^* = 72%, *P* for heterogeneity <0.0001; recessive model: GG+GA vs AA: *I^2^* = 64%, *P* for heterogeneity = 0.001; co-dominant model: GG vs AA: *I^2^* = 69%, *P* for heterogeneity = 0.0002; co-dominant model: GG vs GA: *I^2^* = 72%, *P* for heterogeneity = 0.0001; allelic model: A vs G: *I^2^* = 82%, *P* for heterogeneity <0.00001 and allelic model: G vs A: *I^2^* = 82%, *P* for heterogeneity <0.00001 were noted, respectively (Table 3).

### Publication bias

**Figure 7:**
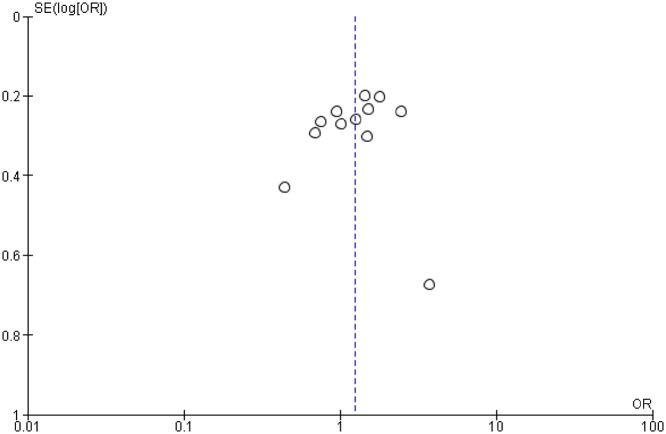
Funnel plot assessing publication bias in recessive model (GG+GA vs AA)

Funnel plots were used in random-effect and fixed-effect models respectively to detect the publication bias. A relatively symmetric distribution in the funnel plot was observed, which indicates that there is no significant publication bias in the included studies (Figure 7).

### TSA

**Figure 8:**
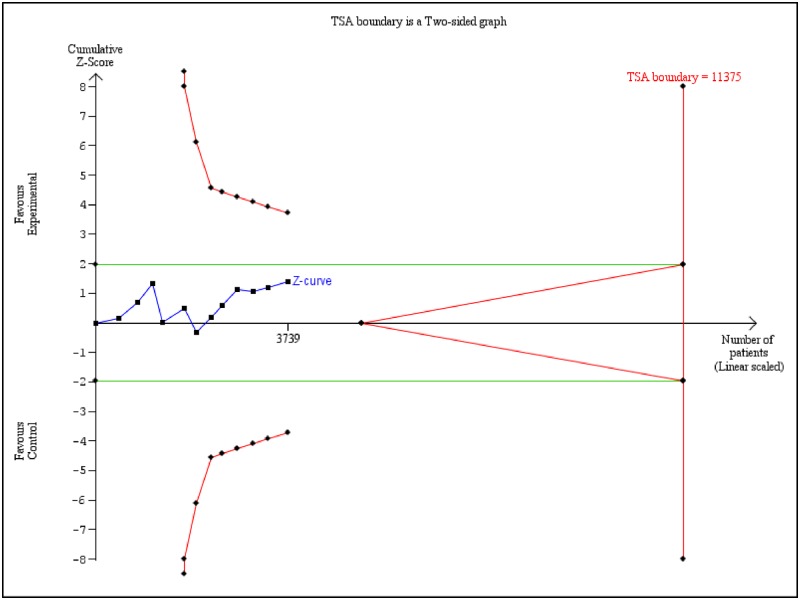
TSA of association of *CCND1* polymorphism (rs9344) and overall cancer risk in Indian population from 12 studies The cumulative Z-curve was constructed by using random-effect model. We calculated α-spending adjusted RIS of 11375 patients using α = 0.05 (two-sided), β = 0.20 (power = 80%). *Note: Z-curve (blue); Conventional boundary (green); TSA boundary (red)*.

The TSA for association between CCND1 polymorphism (rs9344) and overall cancer risk showed that only conventional boundary was crossed by Z-curve, however, it neither crossed the TSA boundary nor the futility area. And the total sample size (3739) did not reach the RIS (11375) (Figure 8). This result indicates that present meta-analysis is inconclusive at this level. Further studies/trials are needed to make this association valid.

## Discussion

*CCND1* is key driver of normal cell cycle regulation and genetic variation in this gene has been reported in many types of cancers. A SNP G870A (rs9344) located on exon-4–intron boundary of *CCND1* has been studied extensively in several cancer types. Several reports from different parts of the world have been published with reference to *CCND1* polymorphism and risk of various types of cancers including cervical, prostate, colorectal, urinary bladder, squamous cell carcinoma of the head and neck etc. [46–50]. Investigators from India also tried to explore the association of CCND1 polymorphism and susceptibility to different cancer types including cervical, breast, oral, esophageal, lung, urinary bladder, prostate, and colorectal [19–29]. However, these reports are conflicting thus we performed meta-analysis on the literature available in order to provide more accurate information on the role of *CCND1* G870A (rs9344) polymorphism and overall cancer risk in Indian population. Although, various meta-analyses on individual cancer susceptibility have been published globally [30–35]. Pabalan et al. (2008) [51], performed a meta-analysis on role of *CCND1* polymorphism in different types of cancers and populations. However, a comprehensive data are lacking from India with overall cancer risk. Hence, we have designed the present study focussed on Indian population.

The present meta-analysis, contained a total of 12 studies comprising 1791 cancer cases and 1948 controls [19–29] showed the lack of significant association between *CCND1* G870A polymorphism (rs9344) and overall cancer risk in all the genetic models. These findings are consistent with the result of another study by Luo et al. (2016) [52], which ruled out the involvement of *CCND1* polymorphism (G870A) with the risk of hepatocellular carcinoma. In the similar lines, study by Zheng et al. (2015) [53] suggested that *CCND1* polymorphism may not be associated with the risk of prostate cancer. Similarly, Wang et al. (2018) [54] also found no significant association between the let-7i rs10877887 and let-7a-1/let-7f-1/let-7d rs13293512 polymorphisms and overall cancer risk. In disagreement with our findings a meta-analysis by Pabalan et al. (2008) [51], showed an increased cancer risk associated with *CCND1*-A870G polymorphism in the human population. Another study by Qin et al. (2014) [55] also indicated that *CCND1* polymorphism may increase the risk of non-Hodgkin lymphoma but it was not true in case of leukemia. On the identical lines, Lin et al. (2014) [56] too observed the lack of association between *CCND1* polymorphism (G870A) and head and neck cancer, however; they found that smokers carrying ‘A’ allele or ‘AA’ genotype for rs9344 SNP located on *CCND1* may be at higher risk to head and neck cancer development.

Our subgroup analysis showed an increased risk (1.52-fold) for cervical cancer development but this association could not attain the limits of statistical significance (*P*=0.38). The possible explanation for this observation may be the small sample size of contributing studies. No promising association of this SNP has been established with the development of cervical cancer in Caucasian population by Yang et al. (2015) [57]. In another study, no significant association was reported between the *CCND1* SNP (rs9344) and overall risk for cervical cancer in the Asian population but on stratification analysis by race, individuals carrying the AA or AA/AG genotypes showed a significant higher risk in comparison with GG carriers [32]. In parallel to the findings from the present study, Hu et al. (2014) [30], also did not find the association of *CCND1* G870A polymorphism and cervical cancer risk amongst different ethnic groups including Asian, Caucasian, and mixed in a cumulative meta-analysis.

Additionally, a significant association between *CCND1* polymorphism and increased risk for breast and esophageal cancer has been established. Similar to our results, Sergentanis and Economopoulos (2011) [58] found that the ‘A’ allele of the *CCND1* G870A polymorphism is associated with higher risk for breast cancer. These findings are further strengthened by another meta-analyses conducted by Lu et al. (2009) [59] and Cui et al. (2012) [60] that showed the association of AA genotype of *CCND1* G870A polymorphism with breast cancer susceptibility. Similarly, Soleimani et al. (2016) [61] showed a significant association between *CCND1* G870A polymorphism and breast cancer risk but in Caucasians. A meta-analysis conducted Wen et al. (2014) [62] supported our data that *CCND1* G870A polymorphism is a potential risk factor in the development of esophageal cancer. Other related meta-analysis by Cai et al. (2013) [63] is not in agreement with our findings and showed lack of potential association between *CCND1* G870A polymorphism and esophageal cancer risk. Likewise, Tang et al. (2015) [64], also observed similar results describing that *CCND1* SNP rs9344 is not having role in esophageal squamous cell carcinoma.The present study suggests that there is a significant correlation between this polymorphism and increased risk of colorectal cancer amongst Indian population. Recently, Xu et al. (2016) [34] suggested that this SNP may increase the risk for developing colorectal cancer with special emphasis to sporadic colorectal cancer in Caucasian population. The study by Jiang et al. (2006) [65] suggested that the *CCND1* G870 AA genotype may increase the colorectal cancer risk compared with the GG+AG genotype (OR = 1.56, 95%CI = 1.10–2.21) in an Indian population. Similarly, Zhang et al. (2016) [33], suggested that *CCND1* polymorphism is a risk factor for gastric cancer in Caucasians. According to the literature search, Dai et al. (2016) [35] also tried to establish the association between *CCND1* polymorphism (rs678653) located on the 3′-UTR and susceptibility to cancer, but they have not studied the polymorphism under investigation G870A (rs9344).

The present study had some limitations, first, all of the included studies were hospital based which may not represent the true population. Second, environmental factors like smoking, use of alcohol, and infections with viruses were not included in the present meta-analysis. Finally, the sample size was reasonably small, which may be the reason for controversial results.

## Conclusion

In conclusion, present meta-analysis showed that *CCND1* SNP (rs9344) may not serve as a risk factor for overall cancer susceptibility in Indian population. However, a significant association between *CCND1* SNP and increased risk for breast, esophageal, and colorectal cancer was found on subgroup analysis. Moreover, a non-significant increased risk for cervical cancer in relation to *CCND1* polymorphism was observed in Indian population. Thus, *CCND1* G870A (rs9344) polymorphism has a potential to be served as a prognostic biomarker for breast, esophageal, and colorectal cancer in Indian population. Still, larger and well-designed studies including other risk factors are warranted in future to validate the findings from present analysis.

## Supporting information

**Table S1:** Quality assessment scoring of included studies in the Meta-analysis.

## Abbreviations

*CCND1*: cyclin D1
OR: odds ratio
PMID: PubMed IDentifier
RevMan: *Review Manager*
RIS: required information size
SNP: single nucleotide polymorphism
TSA: trial sequential analysis
95%CI: 95% confidence interval
