# Supplementary material for: Association between Cyclin D1 G870A (rs9344) polymorphism and cancer risk in Indian population: meta-analysis and trial sequential analysis
Source: Biosci Rep. 2018 Nov 30;38(6):BSR20180694. doi: 10.1042/BSR20180694 (PMC6265616; doi:10.1042/BSR20180694)
Supplement: Supplementary file 1 [file bsr20180694_Supp1.pdf]

Table S1. Quality assessment scoring of included studies in the Meta-analysis.

	Study ID →	16488657	17011980	17561354	18548202	19489683	20380574	21268129	20822933	23354584	24604328	24604328*	25146682
**1.Representativeness of cases**	Criteria Score	1	1	1	1	1	1	1	1	1	1	1	1
Selected from cancer registry or multiple cancer center sites	2												
Selected from oncology department or cancer institute	1												
Not mentioned	0												
**2.Source of controls**		1.5	1.5	1.5	1.5	1.5	1.5	1.5	1.5	1.5	1.5	1.5	1.5
Population or community based	2												
Hospital-based (cancer-free controls without diseases)	1.5												
Healthy volunteers without total description	1												
Cancer-free controls with other system diseases	0.5												
Not mentioned	0												
**3.Ascertainment of relevant cancer**		2	2	2	2	2	2	2	2	2	2	2	2

Histopathologic confirmation	2												
Patient medical record	1												
Not mentioned	0												
**4.Sample size**		1	1	1	1	1	1	1	1	1	0	0	1
>1000	2												
200-1000	1												
<200	0												
**5.Quality control of genotyping methods**		1	0	1	0	1	0.5	1	0.5	0.5	0.5	0.5	0.5
Repetition of partial/total tested samples with a different method	1												
Repetition of partial/total tested samples with the same method	0.5												
Not described	0												
**6.Hardy-Weinberg equilibrium (HWE)**		1	1	1	1	1	1	1	1	1	1	1	1
Hardy-Weinberg equilibrium in control subjects	1												
Hardy-Weinberg disequilibrium in control subjects	0												
	Total	7.5	6.5	7.5	6.5	7.5	7.0	7.5	7.0	7.0	6.0	6.0	7.0
